# Ethers and esters derived from apocynin avoid the interaction between p47*phox* and p22*phox* subunits of NADPH oxidase: evaluation *in vitro* and *in silico*

**DOI:** 10.1042/BSR20130029

**Published:** 2013-08-02

**Authors:** Martha Edith Macías-Pérez, Federico Martínez-Ramos, Itzia Irene Padilla-Martínez, José Correa-Basurto, Lowell Kispert, Jessica Elena Mendieta-Wejebe, Martha Cecilia Rosales-Hernández

**Affiliations:** *Laboratorio de Biofísica y Biocatálisis, Sección de Estudios de Posgrado e Investigación de la Escuela Superior de Medicina del Instituto Politécnico Nacional, Plan de San Luis y Díaz Mirón s/n, Casco de Santo Tomás, México, D.F. 11340, México; †Laboratorio de Química Inorgánica de la Escuela Nacional de Ciencias Biológicas del Instituto Politécnico Nacional, Prolongación de Carpio y Plan de Ayala s/n, Casco de Santo Tomas, México, D.F. 11340, México; ‡Laboratorio de Química Orgánica, Departamento de Ciencias Básicas, Unidad Profesional Interdisciplinaria de Biotecnología del Instituto Politécnico Nacional, Av. Acueducto s/n., Barrio La Laguna, Ticomán, México, D.F. 07340, México; §Laboratorio de Modelado Molecular y Bioinformática, Sección de Estudios de Posgrado e Investigación de la Escuela Superior de Medicina del Instituto Politécnico Nacional, Plan de San Luis y Díaz Mirón s/n, Casco de Santo Tomás, México, D.F. 11340, México; ∥Department of Chemistry, 250 Hackberry Lane, The University of Alabama, Tuscaloosa, AL 35487-0336, U.S.A.

**Keywords:** apocynin derivatives, drug design, inhibitory activity, molecular modelling, NADPH oxidase, 5-ASA, 5-aminosalicylic acid, AIR, autoinhibitory region, AUC, area under the curve, CM-H, 1-hydroxy-3-methoxycarbonyl-2,2,5,5-tetramethylpyrrolidine, DME, dimethoxyethane, DPPH, 1,1-diphenyl-2-picrylhydrazyl, EC, endothelial cell, EPR, electron paramagnetic resonance, MPO, myeloperoxidase, NO, nitric oxide, NOX, NADPH oxidase, PBR, polybasic region, PRR, proline-rich region, SOD, superoxide dismutase, TEA, triethanolamine

## Abstract

NOX (NADPH oxidase) plays an important role during several pathologies because it produces the superoxide anion (O_2_^•−^), which reacts with NO (nitric oxide), diminishing its vasodilator effect. Although different isoforms of NOX are expressed in ECs (endothelial cells) of blood vessels, the NOX2 isoform has been considered the principal therapeutic target for vascular diseases because it can be up-regulated by inhibiting the interaction between its p47*phox* (cytosolic protein) and p22*phox* (transmembrane protein) subunits. In this research, two ethers, 4-(4-acetyl-2-methoxy-phenoxy)-acetic acid (**1**) and 4-(4-acetyl-2-methoxy-phenoxy)-butyric acid (**2**) and two esters, pentanedioic acid mono-(4-acetyl-2-methoxy-phenyl) ester (**3**) and heptanedioic acid mono-(4-acetyl-2-methoxy-phenyl) ester (**4**), which are apocynin derivatives were designed, synthesized and evaluated as NOX inhibitors by quantifying O_2_^•−^ production using EPR (electron paramagnetic resonance) measurements. In addition, the antioxidant activity of apocynin and its derivatives were determined. A docking study was used to identify the interactions between the NOX2′s p47*phox* subunit and apocynin or its derivatives. The results showed that all of the compounds exhibit inhibitory activity on NOX, being **4** the best derivative. However, neither apocynin nor its derivatives were free radical scavengers. On the other hand, the *in silico* studies demonstrated that the apocynin and its derivatives were recognized by the polybasic SH3A and SH3B domains, which are regions of p47*phox* that interact with p22*phox*. Therefore this experimental and theoretical study suggests that compound **4** could prevent the formation of the complex between p47*phox* and p22*phox* without needing to be activated by MPO (myeloperoxidase), this being an advantage over apocynin.

## INTRODUCTION

There are several components in organisms that produce the superoxide anion (O_2_^•−^), the main one is the enzyme NOX (NADPH oxidase) [1–4]. This enzyme is widely distributed in the ECs (endothelial cells) of blood vessels where NOX1, NOX2 and NOX4 are the principal isoforms expressed [5]. However, NOX2 plays an important role during several pathologies due to this isoform being highly up-regulated [6]. NOX2 is a multimeric enzyme whose subunits are located in the cytosol and membrane of resting cells [7]. The p22*phox* and gp91*phox* subunits are transmembrane proteins, whereas p40*phox*, p47*phox*, p67*phox* and the small GTPase *rac*1 are located in the cytosol. It has been shown that the NOX2 isoform from ECs is highly similar to the neutrophil isoform in terms of expression of their subunits p47*phox*, p67*phox*, p22*phox*, gp91*phox* and *rac*1 [8,9].

The activation of NOX implies the translocation of its cytosolic subunits to facilitate the interaction between p47*phox* and p22*phox*, thereby leading to O_2_^•−^ production [10]. An excessive amount of O_2_^•−^ in the vascular tissue has been found in patients who suffer hypertension disease [11–13]. This finding suggests that O_2_^•−^ may be involved in endothelial dysfunction [14–17]. The O_2_^•−^ ion radical reduces the vasodilator properties of NO (nitric oxide), which reacts with high affinity to yield peroxynitrite (ONOO^•−^) as a product [18]. Therefore it has been suggested that NOX2 may be an important therapeutic target for the treatment of hypertension [19]. For this purpose, several strategies have been proposed to inhibit NOX2 catalytic activity [20]. One of the strategies is to prevent the assembly of its cytoplasmic and transmembrane subunits; for that reason, several compounds have been employed. For example, gp91dstat and AEBSF [4-(2-aminoethyl)benzenesulfonyl fluoride] hydrochloride prevent the association of gp91*phox* with p47*phox*, nebivolol prevents the interaction of p67*phox* with *rac1* and PR-39 and the apocynin dimer inhibit the association between p47*phox* and p22*phox* [21]. However, some disadvantages of apocynin use have been reported: (a) apocynin is not able to inhibit NOX because it requires dimerization by MPO (myeloperoxidase), which prevents the interaction with p47*phox* and p22*phox*; (b) when apocynin is administered to rats, a small amount of it is dimerized [21]; and (c) apocynin has been reported as a free radical scavenger instead of as an NOX inhibitor, which could be due to its chemical relatedness to some free radicals scavengers such as 5-ASA (5-aminosalicylic acid) [22].

Owing to pharmaceutical interest in apocynin, the synthesis of amines and imides derived from the compound was recently reported. These compounds were tested for cytotoxicity induced by LPS (lipopolysaccharide). Two of the compounds, one that was chemically similar to the apocynin dimer and another that was a lipoid acid coupled with apocynin, were more efficient than apocynin. These compounds diminish the ROS (reactive oxygen species) content and reduce p67*phox* expression [23]. Moreover, it has been reported that some oligomeric AOP (apocynin oxidation products) produced by soybean peroxidase inhibit NOX activity [24]. Furthermore, the inhibitory activities of other compounds that are chemically related to the apocynin dimer have been reported [25,26]. In addition, the results presented in those works support the theory that apocynin must occur in the dimeric form to be active. These results are of considerable importance because there are tissues that do not have enough MPO (vascular system), and therefore, in these tissues, apocynin cannot be activated. For this reason, it is necessary to synthesize new compounds that can inhibit NOX activity without MPO activation to reduce O_2_^•−^ production.

To accomplish this goal, a set of apocynin derivatives conformed by two ethers, 4-(4-acetyl-2-methoxy-phenoxy)-acetic acid (**1**) and 4-(4-acetyl-2-methoxy-phenoxy)-butyric acid (**2**) and two ester, pentanedioic acid mono-(4-acetyl-2-methoxy-phenyl) ester (**3**) and heptanedioic acid mono-(4-acetyl-2-methoxy-phenyl) ester (**4**), were synthesized and evaluated as NOX inhibitors. Furthermore, the DPPH (1,1-diphenyl-2-picrylhydrazyl) assay and the Fenton reaction were performed to test their antioxidant activities. In addition, a docking study was carried out to show the interactions between p47*phox* and apocynin or its derivatives.

## MATERIALS AND METHODS

### Experimental section

#### Chemistry

All chemicals with the exception of pimelic anhydride were obtained from Sigma-Aldrich and were used without further purification. Pimelic anhydride was synthesized as described [27]. Melting points were determined in open capillary tubes with an ELECTROTHERMAL melting point apparatus. ^1^H NMR and ^13^C NMR spectra were recorded using a Varian Mercury 300 apparatus (^1^H: 300.08, ^13^C: 7.46 MHz). Chemical shifts (δ) were reported in ppm downfield from the internal (CH_3_)_4_Si standard and coupling constants were reported in Hz. IR spectra were assessed using a Spectrum GX FT-IR spectrometer (PerkinElmer). Absorption values are expressed as wavenumbers (cm^−1^); only significant absorption bands are shown. In addition, MS measurements were carried out on an MStation JMS-700 JEOL spectrometer. Reactions were monitored by TLC on aluminium-backed sheets with silica gel 60 GF_254_ (HX805651) and visualized using a UV lamp (254 nm).

#### Synthesis procedure

**Scheme 1:**
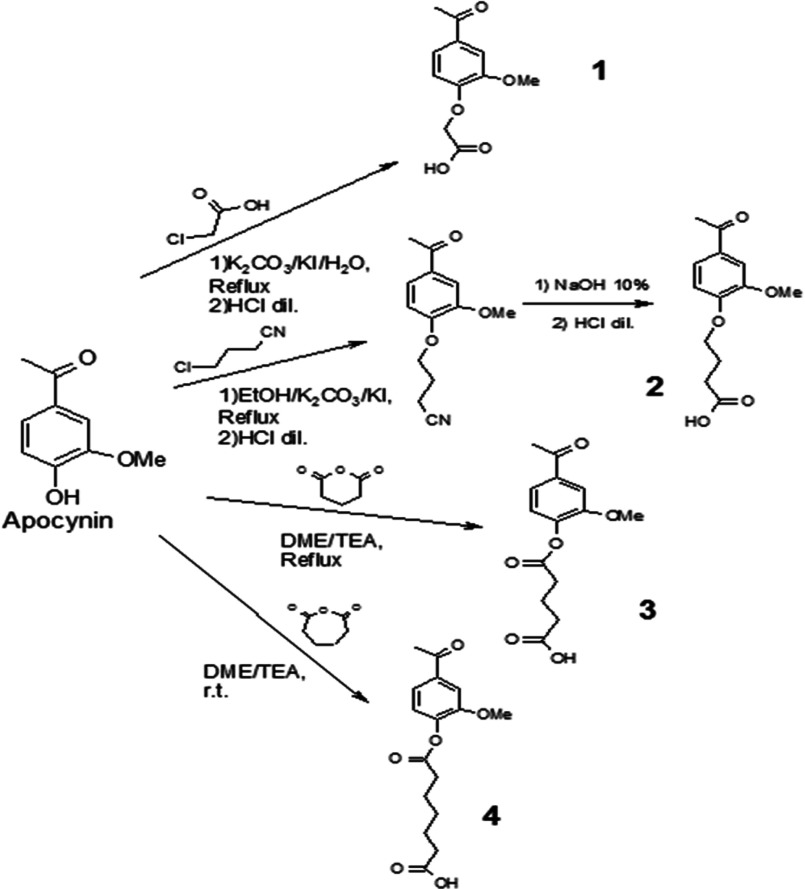
Mechanism of synthesis of apocynin derivatives Synthesis of the ether (**1** and **2**) and ester (**3** and **4**) apocynin derivatives using the starting material apocynin.

The synthesis of apocynin derivatives included several steps depicted in Scheme 1.

(a) *4-(4-Acetyl-2-methoxy-phenoxy)-acetic acid (1)*

Apocynin (166.2 mg (1.0 mmol)) was dissolved in deionized water (10 ml) and 141.8 mg (1.5 mmol) of chloroacetic acid and 166.0 mg (1 mmol) of potassium iodide and 345.5 mg (2.5 mmol) of potassium carbonate were added. The mixture was refluxed for 4 h; the pH was adjusted (neutral) with HCl (1:4 v/v), and the unreacted apocynin and chloroacetic acid were extracted with ethyl acetate (50×30 ml). The aqueous solution was acidified to pH 2, and the resulting precipitate was filtered and dried under vacuum to give 117 mg of compound **1** (50% yield).

White solid: r_f_=0.185 (ethanol/ethyl acetate 9:1); mp=140–142°C; solubility: ethylacetate, DME (dimethoxyethane), DMSO, ethanol; IR: *ν*=3500–2300 (max at 3043, 2769, 2721, 2663, 2563 C-H and O-H st), 1690, 1648, 1284, 1209, 1028 and 1147 cm^−1^ (C–O and C=O st); ^1^H NMR (300.08 MHz DMSO); δ=12.35 (br, 1H, OH), 7.57 (d, ^3^J=8.2, 5-H), 7.44 (s, 1H, 3-H), 6.93 (d, ^3^J=8.7, 6-H), 4.77 (s, 2-H, CH_2_), 3.81 (s, 3H, OCH_3_), 2.51 (s, 3H, COCH_3_); ^13^C NMR (7.46 MHz, DMSO-d6): δ=197.1 (C, C=O), 170.4 (C, COOH), 152.1, 149.2 (2C, C-O), 140.0 (C, 4-C), 123.4 (CH, 5-C), 112.5 (CH, 6-C), 111.2 (CH, 3-C), 65.4 (CH_2_O), 56.2 (OCH_3_), 27.1 (COCH_3_); ESI-MS: m/z 224 [M+1]^+^.

(b) *4-(4-Acetyl-2-methoxy-phenoxy)-butyric acid (2)*

The quantities of 166.2 mg (1.0 mmol) of apocynin and 0.14 ml (1.5 mmol) of 4-chlorobutyronitrile were dissolved in absolute ethanol (5 ml), and 208 mg (1.5 mmol) of potassium carbonate and 190 mg (1.5 mmol) of potassium iodide were added. The mixture was refluxed for 18 h; the solvent was evaporated, and the resulting solid was suspended in 5 ml of water and acidified to pH 4–5 with HCl (1:4 v/v). The remaining apocynin and 4-chlorobutyronitrile were extracted with ethyl acetate (50×30 ml). The aqueous solution was made alkaline with NaOH (10%) and refluxed for 30 min. Finally, the solution was acidified to pH 2 and extracted with ethyl acetate (50×30 ml). The organic phase was dried with anhydrous sodium sulfate and decoloured with activated charcoal. After filtration and evaporation, 128 mg of compound **2** were obtained (51% yield).

Yellow solid: r_f_=0.315 (hexane/ethanol/ethyl acetate 6:2:2); mp=160–162°C; solubility: ethylacetate, DME, DMSO, ethanol; IR: ν=3500–2300 (max at 3000, 2769, 2721, 2663, 2563 C–H and O–H st), 1717, 1588, 1268, 1219, 1155 and 1024 cm^−1^ (C-O and C=O st); ^1^H NMR (300.08 MHz DMSO); δ=12.2 (br, 1H, OH), 7.58 (d, ^3^J=8.2 Hz, 5J=2.1 Hz, 1H, 5-H), 7.42 (d, ^5^J=2.1 Hz, 1H, 3-H), 7.04 (s, ^3^J=8.5, 1H, 6-H), 4.05 (t, ^3^J=6.5, 2H, OCH_2_), 3.80 (s, 3H, OCH_3_), 2.51 (s, 3H, COCH_3_), 2.37 (t, ^3^J=7.3 Hz, 2H, COCH_2_), 1.94 (q, ^3^J=6.9 Hz, 2H, CH_2_); ^13^C NMR (75.46 MHz, DMSO-d6): δ=197.1 (C, C=O), 170.4 (C, COOH), 152.1, 149.2 (2C, C-O), 130.5 (C, 4-C), 123.8 (CH, 5-C), 112.4 (CH, 6-C), 111.0 (CH, 3-C), 68.1 (CH_2_O), 56.2 (OCH_3_), 36.6 (CH_3_C=O), 27.0 (COCH_3_), 24.8 (CH_2_); ESI-MS: m/z 252 [M]^+•^.

(c) *Pentanedioic acid mono-(4-acetyl-2-methoxy-phenyl) ester (3)*

Apocynin (1 mmol) 166.2 mg and 171 mg of glutaric anhydride (1.5 mmol) were dissolved in 10 ml DME, and then 0.153 ml (1.1 mmol) of TEA (triethanolamine) was added. This mixture was refluxed for 1 h with stirring. The DME was evaporated under vacuum, and 30 ml of ethyl acetate was added to redissolve the mixture. TEA was removed with 10 ml of deionized water. The ethyl acetate was dried with anhydrous sodium sulfate and then was evaporated. The resulting solid was dissolved in 20 ml of deionized water, and the pH was adjusted to neutral. Apocynin was extracted with hexane, and then the mixture was acidified with HCl (1:4, v/v) at pH 2 and extracted with ethyl acetate (50×30 ml). The solvent was evaporated under vacuum to yield 174 mg of compound **3** (62% yield).

White solid: r_f_=0.64 (ethanol/ethyl acetate 9:1); mp=70–72°C; solubility: ethylacetate, DMSO and DME; IR: *ν*=3500–2300 (max at 2979, 2825, 2753, 2673 and 2604 C–H and O–H st) 1698, 1602, 1283 and 1130 cm^−1^ (C–O and C=O st); ^1^H NMR (300.08 MHz DMSO); δ=7.60 (d, ^3^J=8.1 Hz, 5-H), 7.57 (s, 1H, 3-H), 7.23 (d, ^3^J=8.1 Hz, 6-H), 3.82 (s, 3H, OCH_3_), 2.57 (s, 3H, OCH_3_), 2.34 (t, ^3^J=7.3 Hz, 2H, OCH_2_), 2.22 (t, J=7.3 Hz, 2H, OCH_2_), 1,83 (q, J=7.3, 2H, CH_2_); ^13^C NMR (75.46 MHz, DMSO-d6): δ=197.7 (C, C=O), 174.8, 171.2 (2C, COOH), 151.6, 143.9 (2C, C–O), 136.3 (C, 4-C), 123.7 (C, 5-C), 122.4 (CH, 6-C), 112.3 (CH, 3-C), 56.3 (OCH_3_), 33.0 (2C, CH_2_C=O), 20.6 (CH_2_); ESI-MS: m/z 280 [M]^+•^.

(d) *Heptanedioic acid mono-(4-acetyl-2-methoxy-phenyl) ester (4)*

Pimelic anhydride 213 mg (1.5 mmol) and 166.2 mg (1.0 mmol) apocynin were dissolved in 5 ml DME, and 0.208 ml of TEA (1.5 mmol) was added. The mixture was stirred for 2 h at room temperature (25°C). Afterwards, 20 ml of water was added, and the TEA was removed using a separatory funnel. The organic phase was dried with anhydrous sodium sulfate, filtered and evaporated. The compound was dissolved in 20 ml of water, pH was adjusted to neutral, and the apocynin extracted with hexane. The mixture was then acidified with HCl (1:4, v/v) to pH 2, and the next day, the compound was crystallized and was washed with hexane (30 ml) to yield 92 mg of compound **4** (30% yield).

White solid: r_f_=0.69 (ethanol/ethyl acetate 9:1); mp=74–76°C; solubility: ethylacetate, DMSO and DME; IR: *ν*=3500–2300 (max at 2952, 2744, 2699 and 2580 C–H and O–H st), 1761, 1699, 1604, 1286, 1128 and 1027 cm^−1^ (C–O and C=O st); ^1^H NMR (300.08 MHz DMSO); δ=7.58 (d, ^3^J=8.1 Hz, 1H, 5-H), 7.57 (s, 1H, 3-H), 7.22 (s, ^3^J=8.1, 1H, 6-H), 3.82 (s, 3H, OCH_3_), 2.58 (s, 3H, COCH_3_), 2.17 (m, 6-H, COCH_2_), 1.44 (m, 6-H, 3-CH_2_); ^13^C NMR (75.46 MHz, DMSO-d6): δ=197.7 (C, C=O), 171.5 (C, 2-COOH), 151.6, 143.9 (^3^C, C-O), 136.3 (C, 4-C), 123.7 (CH, 6-C), 122.3 (CH, 6-C), 112.2 (CH, 3-C), 56.6 (OCCH_3_), 33.7, 28.8, 28.5, 24.8 (CH_2_,1:1:1:2, 5-CH_2_), 27.4 (OCCH_3_); ESI-MS: m/z 308 [M]^+•^.

#### Antioxidant activity evaluation

##### DPPH reduction determination

A 3×10^−5^ M solution of DPPH in DMSO was used, and different concentrations of apocynin (0.013, 0.026, 0.051, 0.102, 0.204, and 0.408 mM) were examined. Furthermore, 5-ASA, which possesses antioxidant activity [28], was included in this experiment, so that comparisons with apocynin and its derivatives could be made. The concentrations of all compounds evaluated during the assay were calculated relative to apocynin. Three different solutions were compared: (a) the compound solution mixed with DMSO (1:1), (b) the compound solution mixed with the DPPH solution and (c) the DPPH solution alone. Finally, the samples were maintained in the dark for 60 min and the absorbance was measured at 590 nm with a Lambda 25 (PerkinElmer) spectrophotometer. The percentage of DPPH reduction was calculated as reported [29].

##### Fenton reaction

The Fenton reaction protocol was as reported by Polyakov [30]. All reagent solutions were prepared immediately before starting the experiment and deaerated by bubbling with N_2_. The final concentration of the compounds used was 0.408 mM. Apocynin derivatives were dissolved in DMSO and then added to a DMSO solution of the spin trap (PBN 10 mM). The concentration of H_2_O_2_ used was 15 mM. The reaction was started by adding 0.5 mM FeCl_2_ suspended in dichloromethane. The solution was transferred to an EPR (electron paramagnetic resonance) capillary tube and the EPR spectrum was recorded. The EPR measurements were carried out at room temperature using a Bruker Biospin’s e-scan spectrometer operating at 86 KHz field modulation. EPR spectra were recorded at X-band frequency (9.728 GHz), 3471.350±59 G field centre and sweep, 21.9 mW microwave power, 0.04 s time constant, 1.10 G modulation amplitude and 2×10^3^ receiver gain [31]. The EPR spectra were recorded in digital form (an average of three scans was used as a working spectrum). The number of paramagnetic species contained in the samples was obtained by double integration of the EPR signals using the WINEPR program; g values were calculated using this program as well.

#### Sample biological preparations

Arteries of 12-week-old male Wistar rats were used. All animals were acclimatized for one week before the experiments. The animals were kept in a controlled temperature and humidity environment with automatic cycles of light and dark (12/12 h). Water and food were provided *ad libitum*. Animals were treated according to the protocol approved by the Ethics and Institutional Animal Care and Use Committees at the Escuela Superior de Medicina-IPN.

The rats were anaesthetized with sodium pentobarbital (50 mg/kg, intraperitoneal). The aorta was removed after thoracotomy and immediately placed in a phosphate solution of pH=7.4 at a concentration of 50 μM. The aorta tissues were homogenized in 950 μl of Tris/HCl buffers at pH 7.4, and 50 μl of protease inhibitor (Complete™, Mini. Cat. No. 11 836 153 001) was added. The samples were centrifuged at 750 ***g*** for 10 min at 4°C using a Universal 320R Hettich centrifuge (1689-A rotor). The supernatant was stored at −80°C until use. The protein concentration was measured using a Cayman kit (Protein Determination Reagent–item no. 704004 and Protein Determination BSA standard–item no. 704 003), which is based on the Bradford method.

#### Catalytic activity of NOX

The aortic homogenized sample was prepared in an inert atmosphere chamber with N_2_, using 30 μg of protein per sample and 10 mM of PMA. These were added to the reaction buffer (sodium phosphate buffer 50 mM, pH 7.4) containing 5 mM of the radical scavenger CM-H (1-hydroxy-3-methoxycarbonyl-2,2,5,5-tetramethylpyrrolidine) and 100 mM of NADH. The measurements were started by the addition of 100 mM of NADPH. The samples were kept in a water bath at 37°C for 50 min. At the end of this incubation period, each sample was placed in a capillary glass (Corning). O_2_^•−^ formation was determined by the oxidation of CM-H (paramagnetic) [32]. Apocynin and its derivatives (10 μM) were added before the addition of PMA to evaluate their inhibitory effects [21,33]. The reaction was performed in the presence of SOD (superoxide dismutase) to make sure that the signal was due to the production of O_2_^•−^. Furthermore, oxypurinol (100 μM) was employed to inhibit xanthine oxidase activity [34].

The EPR measurements were carried out at room temperature using a Bruker Biospin’s e-scan spectrometer operating at 86 kHz field modulation. EPR spectra were recorded at X-band frequency (9.746 GHz), 3480±60 G field centre and sweep, 17.393 mW microwave power, 0.04 s time constant, 52.43 s acquisition time, 1.55 G modulation amplitude and 5.64×10^1^ receiver gain. The EPR spectra were recorded in digital form (an average of three scans was used as a working spectrum). The number of paramagnetic species contained in the samples was obtained by double integration of the EPR signal using the WINEPR software; the *g* values were calculated using this program and DPPH as a marker (*g*=2.0037).

#### In silico evaluations

##### Protein selection

Owing to the importance of the p47*phox* domain during NOX activation, the 3-D (three-dimensional) structures of several p47*phox* segments have been solved by X-ray or NMR studies. These structures are stored in the PDB. For this work, we used 3-D structures of p47*phox* that were the most complete and lacked mutations according multiple alignment studies with sequences retrieved from GenBank (ID: P14598) (1NG2.PDB, 1WLP.PDB, 1OV3.PDB). Two important conformations were used: (a) an autoinhibited form (PDB code: 1NG2) and (b) the p47*phox*–p22*phox* complex (PDB code: 1WLP) [10,35]. These p47*phox* structures possess the PBR (polybasic region) and SH3A and SH3B domains, which are important during NOX activation. Because of this important role, a docking procedure was used to examine these protein regions with the target compounds.

##### Ligand minimization

The ligand minimum energies in 3-D were determined by means of the Gaussian 98 software at the AM1 level [36].

##### Docking procedure

All possible rotatable bonds and partial atomic charges (Gasteiger-Marsili formalism) of the ligands, as well as the Kollman charges for all atoms of the enzyme, were assigned using the AutoDock tool 1.5.4, a program included in AutoDock [37]. Following this, the ligands were docked on the p47*phox* subunit using AutoDock 4.2.0 with the hybrid Lamarckian Genetic Algorithm as the search method, with an initial population of 100 randomly placed individuals and a maximum of 1.0×10^7^ energy evaluations. The resultant docked orientations that were clustered together occurred within an RMSD (root-mean-square deviation) of 0.5 Å. The lowest free energy cluster for each, returned by AutoDock, was used for further analysis, and all other parameters were maintained at the default settings [37]. Three different dockings procedures were performed on the p47*phox* structure (PDB code: 1NG2): the first docking centred on the midpoint of the 1NG2 structure; the second centred on the α carbon of Cys^196^ belonging to the SH3A domain; and the third centred on the α carbon of Trp^263^ belonging to SH3B. It is important to mention that although the docking procedure was performed over each domain, some amino acid residues belonging to other regions of the protein are included due to the conformation of p47*phox*. All docking simulations were performed using a grid box on all proteins (126×126×126 Å) with grid points separated by 0.375 Å. All protein visualizations were performed with VMD (visual molecular dynamics) version 1.9 [38].

### Statistical analysis

The results are presented as the means±S.E. Data were analysed by one-way ANOVA followed by a Holm–Sidak test to determine statistically significant difference (*P*<0.05) between groups. All analyses were performed using the statistical program Sigma Stat for Windows version 2.03 software (SPSS Inc.), and the graphs were realized using the GraphPad Prism version 5.00 software.

## RESULTS AND DISCUSSION

### Chemistry

Ethers **1** and **2** and esters **3** and **4** were synthesized from apocynin (Scheme 1) with the aim of avoiding the inhibitory activity of the apocynin dimers. To form these new derivatives, a hydrocarbonated chain with a carboxyl group was substituted at the apocynin hydroxyl group (–OH). In addition, apocynin has a carbonyl group and a rigid hydrophobic moiety, which allowed us to design compounds with a carboxyl group located at the end of the flexible hydrocarbonated chain to increase the affinity of the compounds for p47*phox*. For the synthesis of the ethers, a nucleophilic aliphatic substitution reaction was used. For instance, compound **1** was obtained by reacting apocynin and chloroacetic acid. Compound **2** was substituted with 4-chlorobutyronitrile. In both cases, the reaction was performed in a basic environment of potassium carbonate and iodine. To obtain the apocynin esters, glutaric and pimelic anhydrides were used. The reaction was started when apocynin was mixed with one of the anhydrides in a TEA and DME solvent. These compounds were purified by extraction with ethyl acetate from neutral aqueous media, followed by solvent evaporation under vacuum. Each compound was characterized using ^1^H and ^13^C NMR, IR and MS. Melting points and yields were determined for all apocynin derivatives that were subjected to *in vitro* evaluations. NMR determinations showed purity ≥98% for compound **1** and ≥97% for compound **2**, whereas for compound **3**, the purity was ≥95% and for compound **4**, it was ≥98%.

### DPPH reduction determinations

**Figure 1:**
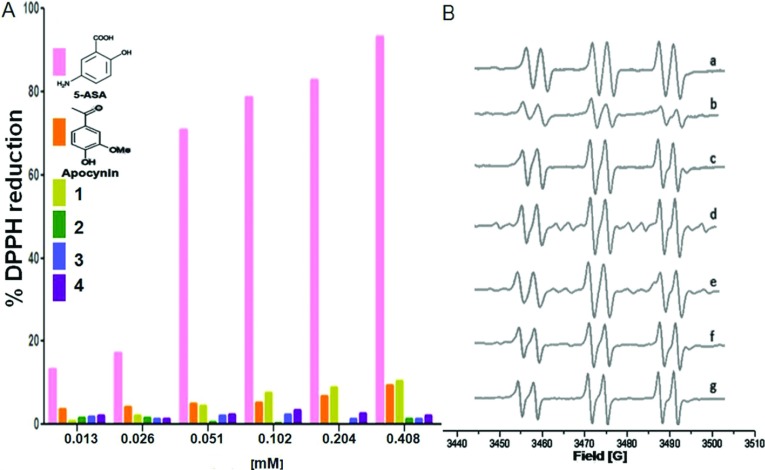
Antioxidant activities of apocynin and its derivatives (**A**) Percent of DPPH reduction at different concentrations of apocynin and its derivatives. The insert in this figure shows the chemical structure of 5-ASA and apocynin. (**B**) EPR spectra of the adduct PBN-CH_3_ formed during the Fenton reaction ([H_2_O_2_]=15 mM, [PBN]=10 mM) (a) without compound, (b) with 5-ASA, (c) with apocynin, (d) with compound **1**, (e) with compound **2**, (f) with compound **3** and (g) with compound **4**.

Figure 1(A) shows the results obtained for the DPPH reduction measurements in which it was observed that neither apocynin nor its ether (**1** and **2**) and ester (**3** and **4**) derivatives showed the ability to reduce DPPH, even when a high concentration was used (0.408 mM). Thus, although apocynin possesses a phenol group similarly to flavonoids, it does not act as a free radical scavenger. This behaviour could be explained based on the substituents in its aromatic ring. As shown in Figure 1(A), apocynin has the electrodonating group (OCH_3_) *ortho* to the hydroxyl group, whereas 5-ASA has an electron-withdrawing group (CO_2_H) in the *ortho* position and an electrodonating group (NH_2_) at the *para* position. Therefore as shown in Figure 1(A), 5-ASA was able to reduce DPPH, contrary to what was observed for apocynin and its derivatives.

### Fenton reaction

The Fenton reaction (Fe^2+^+H_2_O_2_ → Fe^3+^+^•^OH+^−^OH) is used to generate the ^•^OH radical, which at 1 mM H_2_O_2_ reacts with the solvent DMSO to form the methyl (^•^CH_3_) radical. However, if the H_2_O_2_ concentration is increased to 10–15 mM, similar concentrations of ^•^OH and ^•^CH_3_ radicals are formed, whereas at 500 mM, the excess of H_2_O_2_ scavenges the remaining ^•^CH_3_ and ^•^OH, forming ^•^OOH. The spin trap PBN is used to trap the ^•^CH_3_ radical resulting in a characteristic EPR pattern that can be found in the NIEHS database. Examples of the use of the spin trap PBN have been reported by Polyakov et al. [30]. Figure 1B:a shows the EPR spectrum with the hyperfine coupling constants of a_N_=15.14 G and a_H_=3.3 G, which are typically found for the trapping of the ^•^CH_3_ radical and are due to the formation of the PBN-CH_3_ adduct. By monitoring the concentration of the ^•^CH_3_ radical in this way, the short-lived ^•^OH radical can be monitored by noting decreases in the spin-trapped PBN-CH_3_ signal. The same EPR spectral shape was produced in the presence of 5-ASA and apocynin (Figure 1B; b and c, respectively). However, in the presence of 5-ASA, the signal from the adduct diminished in intensity as shown in Figure 1B:b, but a significant difference in the AUC (area under the curve) compared with that in Figure 1B:a was not obtained (results not shown). Therefore 5-ASA was not a good scavenger of the hydroxyl radical; this could have been due to the concentration used (0.408 mM), despite the fact that the higher concentration used in the DPPH assay was also used in this assay. Further EPR measurements showed that neither apocynin (Figure 1B:c) nor its derivatives (Figure 1B:d–g) were good hydroxyl radical scavengers because, although the same concentration was used, a significant difference was not obtained and the PBN-CH_3_ adduct was formed. As shown in Figure 1B:f and g, the signal intensity was not diminished with respect to that shown in Figure 1B:a. These results were in accord with those obtained using DPPH, and we confirmed that apocynin and its derivatives are not free radical scavengers.

### NOX activity

**Figure 2:**
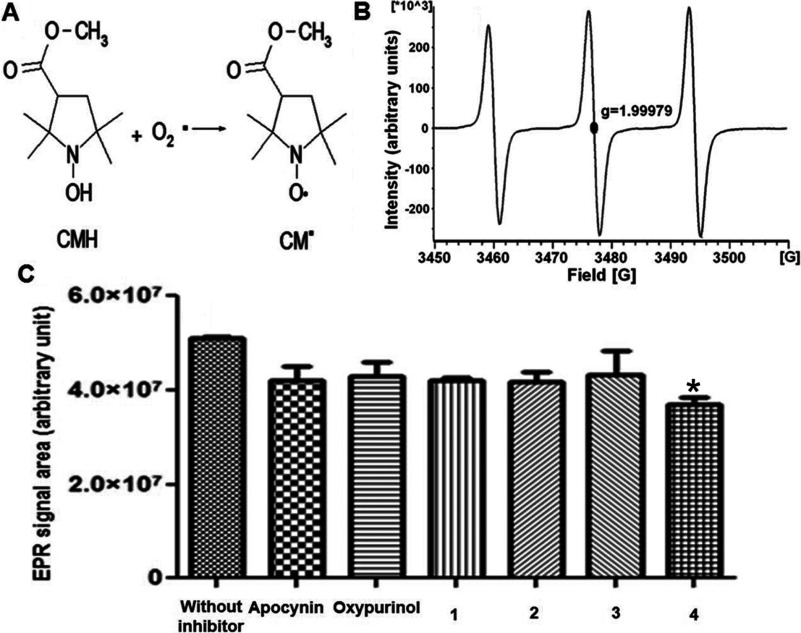
NOX inhibition by apocynin and its derivatives (**A**) Generation of the radical CM^•^ by oxidation of CM-H by the superoxide anion (O_2_^•−^). (**B**) EPR spectrum of the CM^•^ adduct that shows an EPR signal with three lines (*g*=1.99979 with a_N_=17.1 G). (**C**) The EPR signal areas, corresponding to superoxide anion (O_2_^•−^) production by NOX without inhibitor and with oxypurinol, apocynin and its derivatives. *P<0.05: Compound **4** compared with reaction without inhibitor. In agreement with the mechanism proposed for the apocynin dimer (Figure 3), the OH group is necessary to produce the apocynin radical by H subtraction [40]. However, the fact that compound **4** has shown the best activity suggested that the long chain may be important to inhibit NOX.

Inhibitory activities of the apocynin derivatives on NOX were assessed by quantifying O_2_^•−^ production using EPR with CM-H as a spin probe [32]. The spin probe reacted with O_2_^•−^ to form a CM^•^ radical (Figure 2A). This adduct resulted in an EPR spectrum of three lines with *g*=1.99979 (Figure 2B) and a hyperfine splitting constant of a_N_=17.1 G. The area under an EPR signal is directly proportional to the quantity of O_2_^•−^ present in a sample and was measured to determine O_2_^•−^ production by the NOX enzyme [39]. As shown in Figure 2(C) by the increase in area relative to samples without inhibitor, this reaction produced a larger amount of O_2_^•−^. To confirm that the increase in the AUC was due to the production of O_2_^•−^, the SOD enzyme test was employed because this test uses O_2_^•−^ as a substrate to produce H_2_O_2_. Therefore when SOD was added to the reaction, the O_2_^•−^ production decreased (results not shown) as previously reported [32]. These results indicated that the AUC calculated for the reaction corresponded to the O_2_^•−^ production. Consequently, NOX activity was determined in the presence of apocynin or oxypurinol. Figure 2(C) shows that the AUC decreased using either apocynin or oxypurinol; however, these results did not show significant differences with respect to the control.

Comparing the results obtained in this work to those reported by other authors who worked on ECs overexpressing the NOX1, NOX2 and NOX4 [HEK293 (human embryonic kidney cells 293)] isoforms showed that O_2_^•−^ production measured by chemiluminescence did not decrease when apocynin was used. This result is mostly because MPO is not expressed in ECs; therefore apocynin in the dimeric form is not produced. However, in the aortic homogenate used in this work, it is possible that MPO was trapped in the tissue between the muscle cells and the ECs, but that this MPO was not enough to activate a great quantity of apocynin [22].

**Figure 3:**
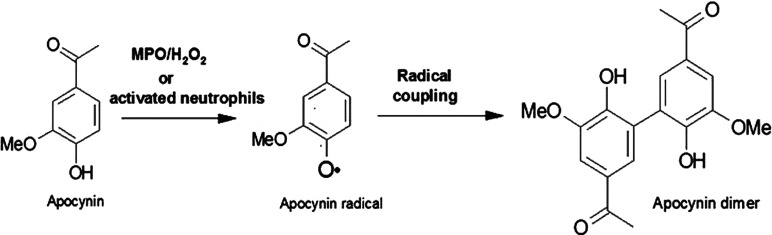
Mechanism of apocynin dimer formation by the MPO enzyme

The apocynin derivatives tested also had inhibitory activity against NOX because O_2_^•−^ production decreased, as depicted in Figure 2(C), which also shows that compound **4** was the best inhibitor (*P*<0.05). Therefore the fact that compound **4** had inhibitory activity against NOX, suggested that the apocynin hydroxyl group was not required for this activity. In addition, by adding hydrophobic and carboxylate groups, it is possible to produce compounds that do not require activation by MPO (Figure 3).

### *In silico* evaluations

**Figure 4:**
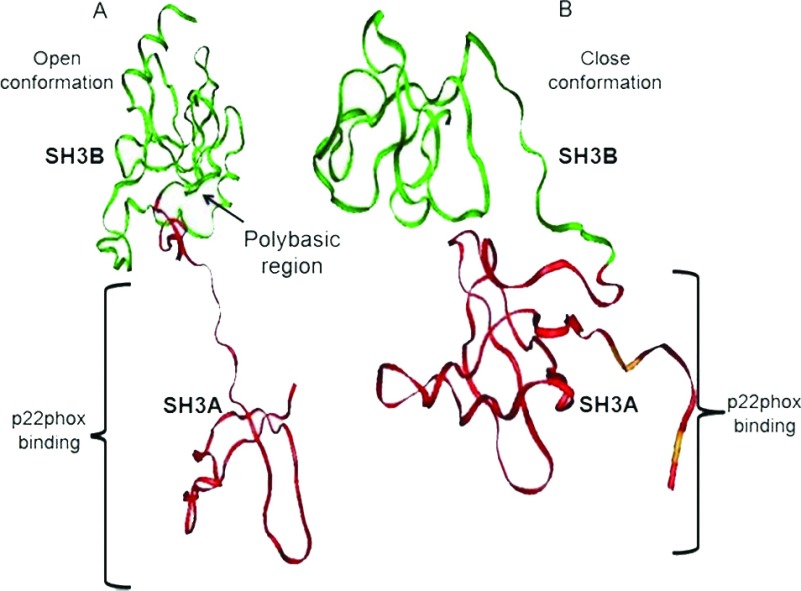
p47*phox* structure (**A**) the p47*phox* crystal segment (PDB code: ING2) of the free protein in an open conformation and (**B**) the p47*phox* crystal segment in the conformation that forms a complex with p22*phox* (PDB code: 1WLP) [35]. The figure only shows the chain that corresponds to p47*phox* that has a globular shape and a closed conformation.

To carry out docking studies, 3-D protein structures are required. However, because complete multimeric complex 3-D structures of NOX are lacking, some segments that recognize apocynin were used [10,35]. One of the most important subunits is p47*phox*, which induces NOX activation by binding to a cytoplasmic region of p22*phox* [41]. The p47*phox* subunit consists of a PX domain (Phox homology domain), two SH3 domains, an arginine/lysine rich region [PBR/AIR (polybasic region/autoinhibitory region)] and a PRR (proline-rich region) (Table 1) [41]. In the inactive state, the tandem SH3 domains interact with the PBR/AIR region (Figure 4A) and, in the activated form, with a PRR from the cytoplasmic domain of p22*phox* (Figure 4B) [7].

**Table 1:** Some properties of the interaction between p47*phox* and apocynin or its derivatives p47*phox* regions and their amino acid residues that interact with apocynin and apocynin derivatives and the free energy (Δ*G*, kcal/mol) values obtained from these interactions. HB, hydrogen bond; π–π:, aromatic interactions; VDW, van Der Waals interactions.

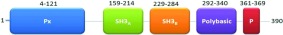									
	∆*G* (kcal/mol)			Amino acid residues involved in the interaction					
Compound	Blind docking	Focused to Cα Cys^196^	Focused to Cα Trp^263^	Blind docking	Main interactions	Focused to Cα Cys^196^	Main interactions	Focused to Cα Trp^263^	Main interactions
Apocynin	−5.13	−5.4		Pro^212^		Cys^196^		Asp^221^	
				Glu^218^		Trp^194^	HB, π-π	Arg^318^	VDW
				Asp^221^		Glu^174^		Glu^218^	HB
				Arg^302^	VDW,HB	Met^175^	HB	Arg^302^	HB,VDW
				Arg^316^	VDW	Tyr^167^	π-π	Arg^316^	VDW
				Arg^318^	VDW,HB	Trp^193^	π-π	Glu^211^	
								Pro^212^	
Apocynin dimer	−6.47	−6.69	−7.01	Trp^204^	π-π	Pro^299^		Asp^221^	
				Pro^206^		Pro^300^		Glu^218^	
				Ala^207^	HB	Pro^206^		Asp^217^	
				Asp^261^		Ile^205^		Pro^216^	
				Arg^296^	VDW:	Trp^204^	π-π:	Glu^211^	
				Gly^297^		Arg^296^	VDW:	Pro^212^	
				Ala^298^		Ala^298^		Lys^317^	VDW
				Pro^299^		Gly^297^	HB	Arg^318^	HB,VDW
				Pro^300^				Arg^316^	VDW
								Arg^302^	VDW
1	−6.13	−5.65	−6.62	Tyr^231^	π-π:	Glu^211^		Ala^290^	
				Lys^258^	HB, VDW:	Pro^216^		Gln^281^	
				Met^278^		Pro^212^		Lys^282^	HB,VDW
				Leu^280^		Arg^316^	HB,VDW	Leu^280^	
				Gln^281^		Glu^218^		Met^278^	
				Lys^282^	HB, VDW:	Leu^210^		Tyr^231^	HB, π-π
				Ala^290^		Ala^207^		Lys^258^	HB,VDW
						Arg^302^	VDW		
2	−6.73	−7.23	−7.33	Met^278^		Trp^264^	π-π	Ala^290^	
				Leu^280^		Lys^258^	HB,VDW	Ile^294^	
				Gln^281^	HB	Tyr^231^	π-π	Met^278^	
				Lys^282^	HB,VDW	Lys^282^	HB,VDW	Ser^277^	
				Gln^285^		Leu^280^		Gln^281^	
				Ala^290^		Ala^290^		Lys^282^	HB,VDW
				Gln^293^		Gln^281^		Tyr^231^	π-π
								Lys^258^	HB,VDW
								Trp^264^	π-π
								Leu^280^	
3	−7.48	−6.99	−6.89	Lys^258^	HB	Ala^290^		Ala^290^	
				Met^278^		Ile^234^		Gln^281^	
				Leu^280^		Gln^281^		Met^278^	
				Gln^281^		Lys^282^	HB,VDW	Leu^280^	
				Lys^282^	HB,VDW	Tyr^231^	HB, π-π	Tyr^231^	HB,VDW
				Ala^290^		Lys^258^	HB,VDW	Lys^282^	HB,VDW
				Gln^293^		Leu^280^		Lys^258^	HB,VDW
						Met^278^			
4	−6.99	−6.94	−7.32	Trp^264^	π-π	Arg^296^	VDW	Lys^258^	HB,VDW
				Ser^277^		Lys^295^	HB,VDW	Trp26	π-π
				Met^278^		Ile^294^		Tyr^231^	π-π
				Leu^280^		Gln^293^		Lys^282^	HB,VDW
				Gln^281^		Ala^290^		Ala^290^	
				Lys^282^	HB,VDW	Gln^281^		Ile^294^	
				Ala^290^		Leu^280^		Leu^280^	
				Gln^293^		Met^278^		Met^278^	
				Ile^294^		Ser^277^		Ser^277^	
				Arg^296^	HB,VDW	Lys^282^	HB,VDW		
						Tyr^231^			

The docking study was conducted in three different forms: (a) the first docking (blind docking) centred on the midpoint of the p47^phox^ segment (PDB code: 1NG2), (b) docking was focused on the amino acid residues belonging to SH3A domain and (c) docking was focused on the SH3B domain (Figure 4).

**Figure 5:**
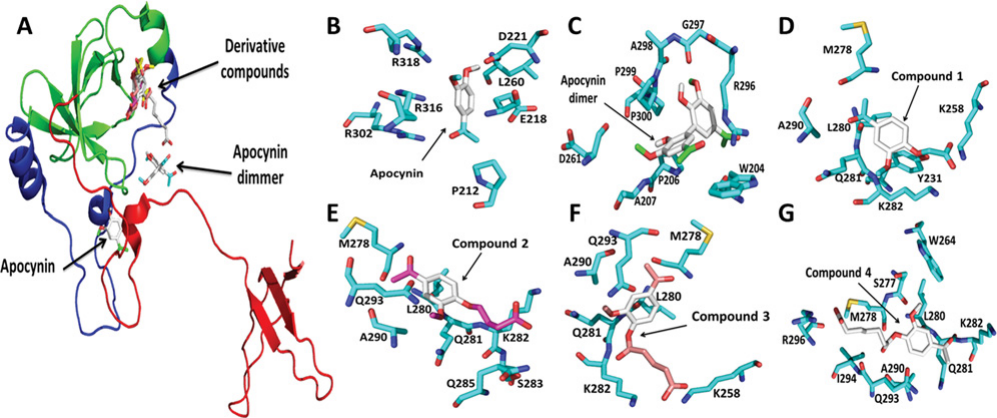
Binding of apocynin, apocynin dimer and apocynin derivatives with the p47*phox* subunit (PDB codes: 1NG2) (**A**) Binding of apocynin and its derivatives with p47*phox* in its open conformation (PDB:1NG2), Interaction of (**B**) apocynin, (**C**) apocynin dimer, (**D**) compound 1, (**E**) compound 2, (**F**) compound 3 and (**G**) compound 4 with p47*phox*. The compounds interact principally with the PBR and the SH3B domain, two important regions that maintain p47*phox* in its autoinhibited form.

The focused docking was performed due to the blind docking does not takes into account the whole protein. In some cases, when docking was focused on one of the two domains, some amino acid residues belonging to the other domain and the PBR were reached. However, despite the fact that different docking methodologies were performed, the compounds tested made interactions with the same amino acid residues (Figure 5A). For instance, apocynin interacted principally with Pro^212^, Glu^218^, Asp^221^, Arg^316^ and Arg^318^, but only some of these residues are involved in the interaction with p22*phox* (Figure 5B; Table 1). However, the apocynin dimer recognized Arg^296^, Gly^297^, Ala^298^, Pro^299^ and Pro^300^ and the region that contains the amino acid residues Trp^204^, Ile^205^, Pro^206^ and Ala^207^ of p47*phox* (Figure 5C). This is of great importance because these regions are part of the PBR (amino acid residues 296–304) and the SH3A domain, respectively (Table 1). As was mentioned before, these structures have important roles in p47*phox* activation. Furthermore, as is shown in Figure 5C, the apocynin dimer interacts with Gly^297^ and Pro^299^, which are crucial to maintaining p47*phox* in its autoinhibited form.

Therefore the apocynin dimer could be inhibiting p47*phox* activity and, consequently, NOX activation by preventing the displacement of the PBR, which blocks p47*phox* binding to p22*phox* in which Pro^299^ is the principal residue involved. In addition, the apocynin derivatives were recognized near the site where apocynin dimer bound and, in some occasions, interacted with the same amino acid residues (Figure 5A). Figure 5 shows the interaction for each compound with p47*phox* when the blind docking was done. Compound **1** (Figure 5D) interact with the SH3B domain and the PBR and the same interactions were observed for compound **2** (Figure 5E) and compound **3** (Figure 5F). However, since the length of the chain of compound **4**, it is bound nearest to the place where apocynin dimer is located (Figure 5G). The specific interaction for each compound with the protein are showed in Table 1.

**Figure 6:**
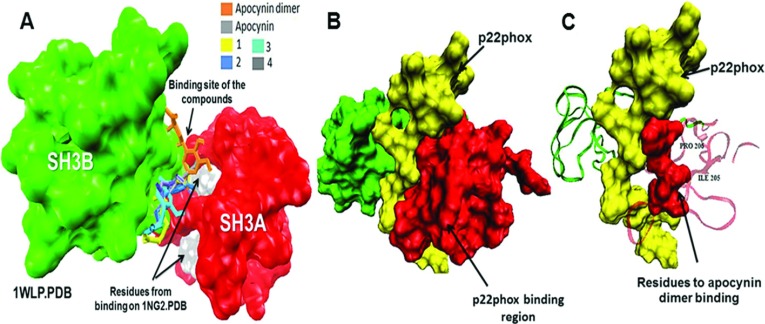
Binding of p22*phox* to p47*phox* (PDB code: 1WLP) (**A**) Binding of apocynin and its derivatives with p47*phox* in its closed conformation (PDB: 1WLP) and (**B**) The p22*phox*–p47*phox* complex. (**C**) Apocynin dimer bound to the P206 and I205 residues of p47*phox* necessary for binding to p22*phox*. It is possible that when this site is occupied by the apocynin dimer, the binding to p22*phox* is prevented.

In addition, as shown in Figure 6(A), the apocynin dimer and apocynin derivatives bound to the same region in the 1WLP structure that participates in the binding of p47*phox* to p22*phox* [35]. In Figure 5(G), is shown the interaction of compound **4** with the PBR and the SH3B domain that are two important regions that maintain p47*phox* in its autoinhibited form. Hence, the apocynin derivatives can inhibit the binding of p22*phox* to p47*phox* by interactions with the SH3A and SH3B domains that prevent complex formation with p22*phox* (Figure 6). Recently, it has been reported that although SH3A is capable of binding to p22*phox*, the affinity of this interaction is significantly increased when both SH3 domains are present [7]. Therefore due to the p47*phox* binding site is formed by two independent domains (SH3A and SH3B), the ligands can recognize different regions of the two SH3 surfaces to bind and maintain p47*phox* in an autoinhibited conformation.

In addition, the apocynin dimer and the apocynin derivatives are not bound to Ser^303^, Ser^304^, Ser^315^, Ser^320^ and Ser^328^ residues that are phosphorylated in activated p47*phox*. However, the binding of the compounds might induce rearrangements and avoid the phosphorylation process due to charge repulsion between the carbonyl groups on the apocynin dimer or the apocynin derivatives and the phosphate group, or the interaction might produce a hindering effect due to the hydrocarbon chain.

Although all the apocynin derivatives were found in the same regions of p47*phox*, the free energy (Δ*G*) values obtained from the interaction between this protein and the apocynin derivatives suggested that compounds **3** and **4** had high affinity during the interaction. In addition, they showed the same amino acid interactions in all docking simulations (Table 1). However, compound **4** was the best inhibitor during *in vitro* assays, which was also corroborated by the docking simulations.

Compound **4** was the best NOX inhibitor due to its ability to reduce the production of O_2_^•−^ as determined by EPR measurements. This compound does not have a free hydroxyl group on the apocynin aromatic ring, so it is not able to form a dimer. Therefore to show inhibitory NOX activity, MPO activation of this compound is not necessary, which is a great advantage in comparison with apocynin. In addition, using the DPPH assay and the Fenton reaction, it was determined that compound **4** is not a free radical scavenger. The *in silico* studies showed that compound **4** contacts the PBR and the SH3B domain, which are important regions for maintaining p47*phox* in its autoinhibited form and preventing interactions with p22*phox*. In conclusion, the use of this compound allows increased bioavailability of NO for increased vascular relaxation.
